# A Comparative Study on Anticancer Effects of the *Alhagi maurorum* and *Amygdalus haussknechtii* Extracts Alone and in Combination with Docetaxel on 4T1 Breast Cancer Cells

**DOI:** 10.1155/2021/5517944

**Published:** 2021-06-14

**Authors:** Nayereh Bahamin, Shahin Ahmadian, Mahmoud Rafieian-Kopaei, Gholamreza Mobini, Mahshid Shafiezadeh, Amin Soltani

**Affiliations:** ^1^Department of Biochemistry, Institute of Biochemistry and Biophysics (IBB), University of Tehran, Tehran, Iran; ^2^Medical Plants Research Center, Basic Health Sciences Institute, Shahrekord University of Medical Sciences, Shahrekord, Iran; ^3^Cancer Research Center, Shahrekord University of Medical Sciences, Shahrekord, Iran

## Abstract

Medicinal plants have long been studied due to their anticancer effects and use of them is commonly increased as a complementary and alternative medicine (CAM therapies) among patients with cancer. In this study, *Alhagi maurorum* (A.m) and *Amygdalus haussknechtii* (A.h) extracts were evaluated for their effects on inhibiting the growth of 4T1 breast cancer cells. Based on MTT assay results, the IC50s of A.m and A.h extracts were 57 *µ*g/ml and 85 *µ*g/ml, respectively. Then the cell migration, gene expression, and degree of apoptosis after 48 hours in each treated group with A.m and A.h extracts alone or in combination with docetaxel (DTX) on 4T1 cells were evaluated. A.m had a synergistic behavior with DTX (CI < 1). A.h reduced DTX IC50 but presented CI > 1. Cell migration assay showed that each extract alone or in combination with DTX prevented the migration of 4T1 cells. The Ao/EB staining and flowcytometry results confirmed that, in combination therapy, A.m + DTX and A.h + DTX induced apoptosis close to the level of DTX. Real-time PCR analysis showed that A.m + DTX (IC50 + IC25) downregulated the mRNA expression of HIF-1*α* and FZD7. A.m + DTX (IC50 + IC10) group decreased the expression of HIF-1*α*. Moreover, in A.h + DTX (IC50 + IC25) group, *β*-Catenin and FZD7 were downregulated and upregulated, respectively. Generally, our findings suggest that the combination of A.m and DTX possesses synergistic antitumor effects on 4T1 cells, which may be a valuable choice for CAM therapies. A.h has an acceptable antitumor activity but not in combination with DTX.

## 1. Introduction

Despite enormous medicinal developments in recent decades, a large number of people suffer from cancer. Based on the statistics adapted from the American Cancer Society and the International Union Against Cancer, 27 million diagnoses and 17 million deaths will occur by 2030 worldwide [1]. Breast cancer is the first deadly cancer among women [2]. Triple-negative breast cancer (TNBC) is one of the breast cancer subtypes that cannot be controlled with standard-of-care therapy [3].

Chemotherapy drugs usually have a lot of side effects and limitations due to nonspecificity, and resistance to some of them is usually observed sometime after using the drug [4–6]. Docetaxel (Taxotere®) (DTX) is a semisynthetic derivative of paclitaxel which was found to be more effective because of being soluble in water. DTX is used as a treatment remedy in patients suffering from breast, ovarian, and/or metastatic cancers [7]. Today, studies have shown that many plants recommended in traditional medicine against cancer have positive effects on cell lines in laboratory or animal studies and they are great sources for discovering new drugs.

The concurrent use of multiple medications can apply treatment from multiple paths to one or more targets. Combination therapy or synergistic therapy has several benefits such as increasing treatment efficacy, reducing dose and toxicity, and minimizing drug resistance. Hence, drug combinations have been widely used, especially in most dreadful diseases, such as cancer, and infectious diseases, including acquired immunodeficiency syndrome (AIDS) [8]. Also, combining medicinal plants with chemotherapeutics, including DTX, can be useful in reducing the toxicity and side effects of chemotherapy [6, 9].

One of the pathways which is tightly associated with cancer and is activated in more than 50% of breast cancers is Wnt signaling [10]. This pathway consists of *β*-Catenin dependent and independent signaling groups [11]. In many studies, it has been shown that a number of medicinal plants or their compounds are effective on the activity of the Wnt path [12–14]. The use of complementary and alternative medicines (CAM) in cancer patients is growing, and pharmacokinetic interactions between CAM and chemotherapeutics that might lead to either increased or decreased plasma levels of anticancer drugs should be investigated [15].

Before starting the main study, we found the effective plants in the treatment of various types of cancer from literature to find the most effective and desired plants and then selected 20 of the available types. Then we tested their inhibitory effect on the growth of 4T1 breast cancer cells, among which A. m and A. h had the most favorable effect, respectively (data are available in the supplementary file).

*A. maurorum* is a plant from the Fabiaceae family which has been widely used in traditional medicine in the treatment of various diseases, and scientists have recently studied its anticancer effect on some cell lines [16–18]. This plant has many biological properties such as antioxidant, antimicrobial, cytotoxic, anti-inflammatory, antiproliferative properties [19]. Previous studies revealed the presence of flavonoids, glycosides, saponins, tannins, alkaloids, steroids, and anthraquinone in the A. m extract [20, 21].

Also Lupeol as an active fraction was detected by nuclear magnetic resonance in A. m inducing apoptosis in human breast cancer cells [22].

*A. haussknechtii* is one of the amygdalus species which is grown in a few provinces of Iran, including Chaharmahal and Bakhtiari. During local research, we found that indigenous people, in addition to using the fruit of this plant, use its leaves to treat some diseases. Therefore, we decided to examine it in this study. In a study, the phenolic content of leaves of several types of wild almonds was examined and it was found that the highest phenolic content (gallic acid) was related to A. h [23]. Because of the benefits mentioned above for combination therapy, the aim of the current study was to evaluate the effects of A. m and A. h extracts alone and in combination with docetaxel on 4T1 breast cancer cells.

## 2. Experimental

### 2.1. Cell Culture

4T1 cells were obtained from the Pasteur Institute (Tehran, Iran). The 4T1 cells were cultured in RPMI1640 medium containing 10% Fetal Bovine Serum (FBS) and 1% Penicillin-Streptomycin and were kept in a humidified incubator at 37°C with 5% CO_2_.

FBS (Gibco, Auckland, New Zealand), RPMI1640 medium, Penicillin-Streptomycin, MTT ([3-(4,5-dimethylthiazol-2-yl)-2,4-diphenyl tetrazolium bromide]) (Sigma, St. Louis, MO, USA), DMSO (dimethyl sulphoxide) (Merck, Hohenbrunn, Germany), and PBS (Sigma-Aldrich, Steinhem, Germany) were used in cell culture and cytotoxic activity evaluation.

### 2.2. Medicinal Plants and DTX

A. h leaves were collected from Chaharmahal and Bakhtiari province and aerial parts of A. m were purchased from authentic local store and confirmed by a botanist at the Medical Plants Research Center, Shahrekord University of Medical Sciences, Shahrekord, Iran. The plants were dried in shadow and grounded.

DTX (20 mg) manufactured by NanoAlvand Co. (Tehran, Iran) was purchased from a drugstore.

### 2.3. Plant Extraction

Ethanol (Merck, Hohenbrunn, Germany) was used for plants extraction. Hydroalcoholic extract (ethanol 70%) was prepared and maceration method was used for plants extraction. To this purpose, the dried powdered plants were soaked in a hydroalcoholic solution for 48 hours, and, after passing through the filter paper, they were concentrated with a rotary machine and the solvent was removed. The concentrated extracts were dried in freeze dryer and the resulting powders were kept at 2–8°C for future assays. The extract powders were dissolved in DMSO to make the stock solutions of each sample (5 mg mL^−1^). Serial dilutions were made from the stock solutions to reach final concentrations (DMSO 1%) [24].

### 2.4. Assessment of Cell Growth after Treatment

For quantification of cell viability using MTT assay, after trypsinization of 4T1 cells, they were seeded onto 96-well plates at 6 × 10^5^ and incubated at 37°C. After 24 h of incubation and when the cells had about 35% confluences, they were treated with fresh medium containing serial dilutions of each plant extract. The control cells medium also contained 1% DMSO vehicle. After 48 h of treatment, the supernatants were removed and MTT solution (0.5 mg mL^−1^) was added and incubated for 4 h. Then, the supernatants were removed, and DMSO was added. The plates were then placed on a shaker for 20 minutes. The absorbance of each well was measured in Stat Fax2100 microtiter plate reader at the wavelength of 570 nm. In this way, cell survival was measured in the form of [A] samples/[A] control*∗*100, where [A] was the absorbance of test samples and control, respectively. The dose-viability curves were fitted by Excel software and IC50 values were calculated. In such studies, the extracts with IC50 greater than 100 *μ*g/mL were considered as ineffective extracts [24]. All experiments were carried out in 3 individual plates with at least three parallel wells.

### 2.5. Assessment of Combined Treatment Using Median-Effect Plot Analysis

CompuSyn software (set up by Dr. Dorothy Chou, ComboSyn Inc., 2005) was used for analysis of results of the MTT assay. Briefly, dose-response curves were generated for individual drugs, and then combination indexes (CI) were calculated for each combination. CI < 1 represents synergy, CI = 1 shows additive effect, and CI > 1 indicates antagonism.

### 2.6. Apoptosis Analysis Using Annexin V Staining

Double-staining was done using the Annexin V and Propidium Iodide kit **(**BD Pharmingen™**)** according to the manufacturer's protocol in order to determine percentages of viable, apoptotic, and necrotic cells following 48-hour exposure to DTX, A. m and A. h extracts, and combination of each extract with DTX. For this purpose, 4T1 cells were seeded onto 6 well plates, and, after treatment with each of the above items, they were trypsinized with repetitive pipetting and washed with PBS (centrifuged at 1200 rpm for 5 min). Untreated cells were used as negative controls. After counting them, they were resuspended in 100 *μ*l of the binding buffer solution per 100000 cells. After adding annexin V and PI, they were incubated for 15 min in the dark at 25°C. Annexin V-FITC binding was detected by flow cytometer (space Partec, Germany) (Ex = 488 nm, Em = FITC 495–519 nm, PI 535–617 nm, FL1 filter for annexin V/FITC and FL2 filter for PI). The data were analyzed using FloMax software version 2.70 (Partec GmbH, Münster, Germany).

### 2.7. Acridine Orange/Ethidium Bromide (AO/EB) Staining

Acridine Orange/Ethidium Bromide (AO/EB) was purchased from Sigma Chemical Company (Becton Dickinson, San, CA, USA). This test is done to visualize nuclear changes and apoptotic body formation and observation of characteristics of apoptosis. Cells were viewed under Zeiss fluorescence microscope connected to a CCD camera. Briefly, 4T1 cells were seeded onto 6-well plates and treated for 48 hours with DTX, A. m and A. h extracts, and combination of each extract with DTX. Then, they were harvested and 25 *µ*l of cell suspension (0.5 × 10^6^ to 2.0 × 10^6^ cells/ml) was incubated with 1 *µ*l of AO/EB solution. Each sample was mixed just prior to microscopy. Then, 10 *µ*l of cell suspension was placed onto a microscopic slide, covered with a glass coverslip, and examined using a fluorescein filter and a 40X objective.

### 2.8. Cell Migration Assay

The 4T1 cells were seeded onto 12-well culture plates to form a monolayer, and then identical straight strip scratches were created using sterile yellow tips. After that, the cells were washed with PBS and incubated with RPMI containing 2% FBS (this FBS concentration allows cell survival with no cell proliferation) and diﬀerent concentrations of hydroalcoholic extract of A. m (IC10), A. h (IC10), and DTX (IC10) and the combination groups of each of these two extracts with DTX (IC10 of DTX with IC10 extracts). After 48 h of incubation, the 4T1 cells were washed with PBS and photographed using a camera connected to an inverted microscope (Nikon, Eclipse TS100) at 10× magnifications. Relative width of scratch was obtained using ImageJ software.

### 2.9. Quantitative Real-Time PCR

Total RNA was extracted from 4T1 cells treated with each of A. m, A. h, and DTX as well as A. m and A. h combined with DTX (IC50 of each of extracts with IC10 and IC25 of DTX) for 48 h using the total RNA extraction kit (Pars Tous, Iran). The quantity of the isolated RNA was characterized by NanoDrop ND-1000 (NanoDrop Technologies, Wilmington, DE). The complementary DNA was synthesized by cDNA synthesis kit (Pars Tous, Iran). Primers for genes, frizzled7 (FZD7), hypoxia inducible factor 1*α* (HIF-1*α*), vascular endothelial growth factor-a (VEGF-a), beta-Catenin (*β*-Catenin), and the housekeeping gene glyceraldehydes 3-phosphate dehydrogenase (GAPDH) were designed using Oligo7 software (Table 1). SYBR Premix Ex Taq^TM^ II (Tli RNase H Plus) (Takara BIO, Inc., Otsu City, Shiga, Japan) was used. Real-time PCR amplifications were run using a Rotor-Gene 3000 (Corbett Research, Sydney, Australia). Normalization of the expression levels of the target genes to the housekeeping gene GAPDH was done using REST software (QIAGEN GmbH, 2009, v2.0.13). Finally, the fold change in expression level of each mRNA was quantified based on Pfaffl method.

### 2.10. Statistical Analysis

Experimental data were expressed by mean ± standard deviation of three independent assays. The results of gene expression were shown as mean ± standard error of the mean.

Statistical significance was calculated using one-way ANOVA by GraphPad Prism software. Statistically different values were defined to be significant at  ^*∗*^*P* < 0.05,  ^*∗∗*^*P* < 0.01,  ^*∗∗∗*^*P* < 0.001,  and ^*∗∗∗∗*^*P* < 0.0001.

## 3. Results

### 3.1. Effects of A.m and A.h on Viability of 4T1 Cells

**Figure 1:**
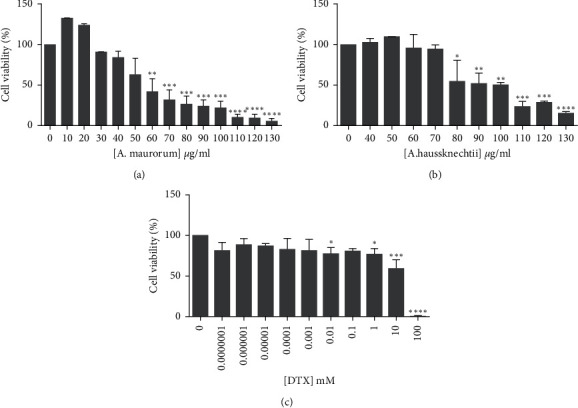
Effect of DTX and A.m and A.h extracts on viability of 4T1 cell line. (a) 4T1 cells treated with varying doses of A.m for 48 h. (b) 4T1 cells treated with varying doses of A.h for 48 h. (c) 4T1 cells treated with varying doses of DTX for 48 h. Cell viability was determined using MTT assay. Results are reported as a percentage of viability compared with control and are presented as mean ± SD from three independent experiments. Significance was set at  ^*∗*^*P* < 0.05,  ^*∗∗*^*P* < 0.01,  ^*∗∗∗*^*P* < 0.001 and ^*∗∗∗∗*^*P* < 0.0001.

MTT assay showed that treating 4T1 cells with different concentrations of each of the mentioned medicinal plants caused various reductions in cell viability. The IC50 values were about 57 and 85 *µ*g/ml for A. m and A. h, respectively.  Figure 1 shows the effect of A. m and A. h extracts on viability of 4T1 cell line. In the following, the meanings of the cases referred to by IC10, IC25, and IC50 are the following numbers; for A. m: IC10 = 32 *µ*g/ml, IC25 = 40 *µ*g/ml, and IC50 = 57 *µ*g/ml; for A. h: IC10 = 10 *µ*g/ml, IC25 = 33 *µ*g/ml, and IC50 = 85 *µ*g/ml; and for DTX: IC10 = 0.4 *µ*M, IC25 = 3.5 *µ*M, and IC50 = 11 *µ*M.

### 3.2. Combined Effect of A.m and A.h Extracts with DTX on 4T1 Cells

A. m and A. h extracts both showed IC50 < 100 *µ*g ml^−1^ in 4T1 cells. This represents very good potential of these plants to inhibit the growth of this cancer cell line. Since some medicinal plants have been shown to have synergistic effects in combination with chemical drugs or they have been able to reduce their side effects or drug resistance [9, 25, 26], we investigated the combination of these two plants with DTX, which is used to treat breast cancer. First, using MTT assay, the IC50 of DTX was calculated to be about 11 *µ*M (Figure 1(c)). Then, by selecting some concentrations lower and higher than IC50 for each extract or DTX, MTT assay was done again in combination mode. The combination index (CI) was calculated by entering doses and their effects in CompuSyn software.

**Figure 2:**
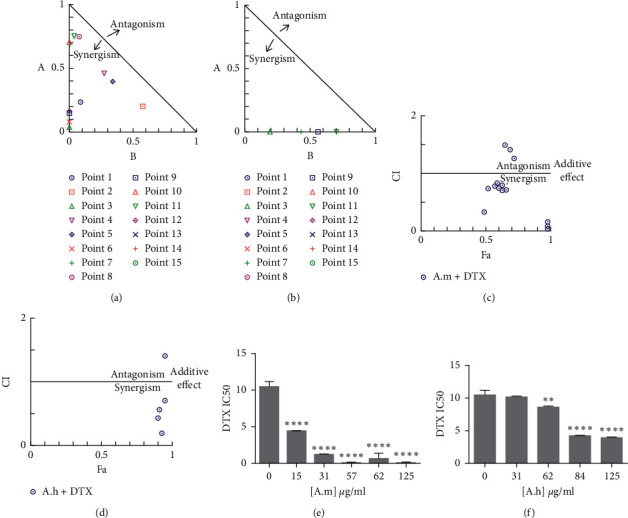
Combination effect of DTX and A.m or A.h extract on 4T1 cells. ((a) and (b)) Isobologram analysis represents the synergistic effect of most of points for A.m + DTX combination. A.m concentrations > 100 *µ*g/ml in combination mode represent antagonism. The combination index (CI) was calculated by CompuSyn software. Points above and below the isoeffect line reflect antagonism and synergy, respectively. 15 data points were entered for each combination. ((c) and (d)) The fraction affected (FA) versus CI curve: FA is defined as the growth inhibition affected by the dose. CI < 1, CI = 1, and CI > 1 indicate synergism, additive effect (solid line), and antagonism effects, respectively. (e) Effect of A.m extract on the IC50 value of DTX : 4T1 cells were treated with A.m + DTX at various concentrations for 48 h and then cell viability was assessed by MTT assay. Combined treatments resulted in significant decrease in IC50 of DTX at 15, 31, 51, 62, and 125 *µ*g/ml of A.m. (f) Effect of A.h extract on the IC50 value of DTX: 4T1 cells were treated with A.h + DTX at multiple concentrations for 48 (h) and then cell viability was assessed by MTT assay. Combined treatments resulted in a significant decrease in IC50 of DTX at 62, 84, and, 125 *µ*g/ml of A.h. Values are given as mean ± SD of three independent experiments.  ^*∗∗∗∗*^*P* < 0.0001,  ^*∗∗*^*P* < 0.01 represent significant changes in the IC50 of DTX.

According to the calculations performed by software, it was found that A. m at doses below 100 *µ*g/ml in combination with DTX had a CI < 1, indicating synergy. The results of isobologram analysis (Figure 2(a)) were in agreement with fraction affected (FA) versus CI; in this way A. m at doses below 100 *µ*g/ml in combination with DTX was below the line of additive effects, demonstrating the synergistic antiproliferative effect of DTX + *A*. m combination (Figure 2(c)). A. m concentrations above 100 *µ*g/ml had antagonistic effects. The results of combining A. h extract with DTX showed that most of the points were above the additive effect line and had antagonistic effects. These results of isobologram analyses and FA versus CI are shown in Figures 2(b) and 2(d), respectively.  Table 2 shows the doses used for extracts and DTX, as well as the values of CI obtained from the software analysis for each combination.

In other experiments, we investigated the effects of the increased concentrations of A. m extract on a range of DTX concentrations (15, 31, 57, 62, and 125 *µ*g/ml of A. m extract and 31, 62, 84, and 125 *µ*g/ml of A. h extract each time in a separate trial). Results showed that A. m significantly reduced the IC50 values of DTX, but A. h showed less effect (Figures 2(e) and 2(f)).

### 3.3. Effects of A.m and A.h Extracts on Migration of 4T1 Cells

**Figure 3:**
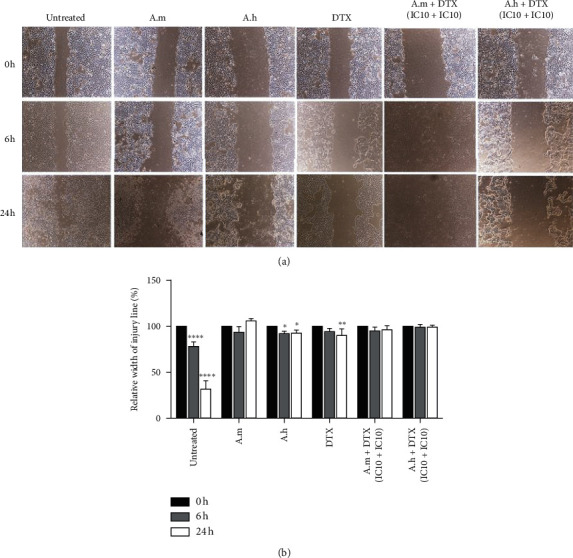
Effect of DTX and A.m and A.h extracts on 4T1 cell migration. (a) Images of the cell migration assay of 4T1 cells following incubation with DTX (IC10), A.m (IC10), A.h (IC10), DTX + A.m (IC10 + IC10), and DTX + A.h (IC10 + IC10) or without treatment, at 0, 6, and 24 h. (b) Quantitative analysis of the scratch assay based on the width of scratch at 0 h. Relative widths at 6 and 24 h were calculated and presented as the mean ± SD from three independent experiments. Significance was set at ^*∗*^*P* < 0.1,  ^*∗∗*^*P* < 0.01,  ^*∗∗∗∗*^*P* < 0.0001. IC10 values for A.m, A.h, and DTX were 32 *µ*g/ml, 10 *µ*g/ml, and 0.4 *µ*M, respectively.

As shown in Figure 3, in treated groups, the scratch closure was delayed after 24 hours. At time 0, the relative width of injury line (scratch width) in the untreated group and in the other treated groups is considered 100%. In the untreated group, after 12 and 24 hours, significant scratch closure was observed due to the migration of 4T1 cells. In the group treated with A. m (IC10), the relative width of the scratch did not change much after 24 hours. A. h (IC10) yielded a relative width of ∼92.68% and DTX (IC10) had a relative width of about 90.13% after 24 h. In the group treated with A. h + DTX (IC10 of both of them) and A. m + DTX (IC10 of both of them), the scratch remained approximately the same after 24 hours with relative width of 99.48% and 96.14%, respectively, but the cells were a little deformed and some of the cell debris was seen in the gap. These results indicate that the used treatments can significantly impede the migration of 4T1 cells. In the untreated group, the gap was significantly closed, but, in other groups, except for DTX and A. h, which slightly reduced the scratch width, there was no significant difference across the scratch after 24 hours.

### 3.4. Apoptosis Analysis after Treatment with DTX, A.m, and A.h Extracts Alone or in Combination with DTX in 4T1 Cell Line

**Figure 4:**
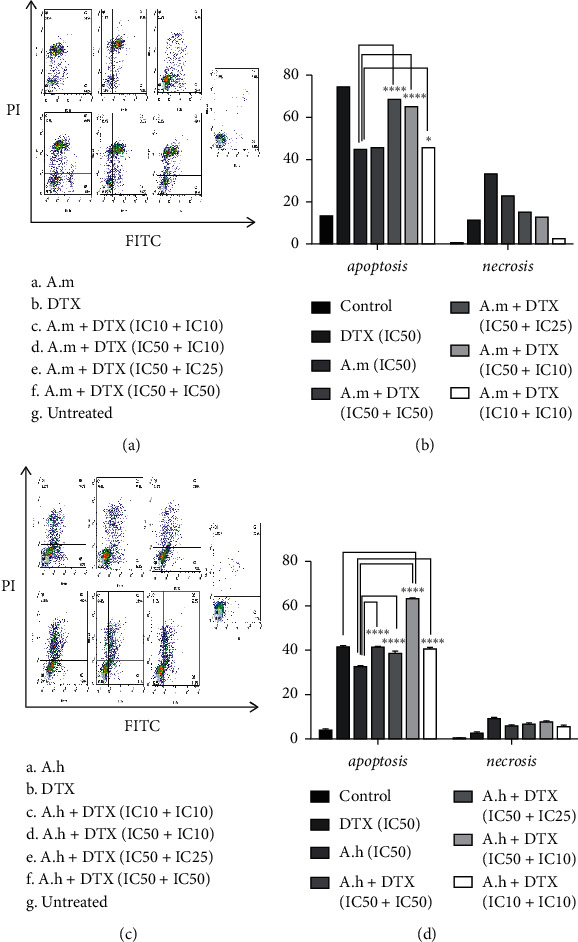
Flow cytometric analysis of apoptotic detection. (a, b) Effect of A.m and DTX alone and in combination with each other on induction of apoptosis of 4T1 cells. A.m + DTX (IC50 + IC25; IC50 + IC10; IC10 + IC10) combination groups had a bigger apoptotic effect compared to A.m extract alone. (c, d) Effect of individual and combinational A.h and DTX on induction of apoptosis of 4T1 cells. All combination groups had a bigger apoptotic effect compared to A.h extract alone, and A.h + DTX (IC50 + IC10) group had a bigger apoptotic effect compared to both of A.h and DTX alone. IC values were as follows: for A.m : IC10 = 32 *µ*g/ml, IC25 = 40 *µ*g/ml, and IC50 = 57 *µ*g/ml; for A.h : IC10 = 10 *µ*g/ml, IC25 = 33 *µ*g/ml, and IC50 = 85 *µ*g/ml; and for DTX : IC10 = 0.4 *µ*M, IC25 = 3.5 *µ*M, and IC50 = 11 *µ*M.

In the case of A. m, IC50 of this extract significantly induced apoptosis (45.12%) compared to control group (13.53%). But A. m + DTX (IC50+IC25) and A. m + DTX (IC50 + IC10) groups increased apoptosis (68.92% and 65.5%, respectively) to a level close to DTX (74.85%) and this effect was significantly higher than that of A. m alone. A. m + DTX (IC10 + IC10) group (46.2% apoptosis) had the least necrosis. A. h + DTX (IC10 + IC10) (40.8%) increased the extent of apoptosis close to the amount of DTX (41.6%). A. h + DTX (IC50 + IC10) group (63.3%) increased apoptosis even more than DTX alone. In all groups, apoptosis was increased significantly compared to the control. The obtained results are shown in Figure 4.

### 3.5. AO/EB Staining Assay for Detection of Apoptotic Cells

Apoptotic and necrotic cells are detectable under fluorescence microscope based on the cell morphology and membrane integrity. By penetrating into viable cells, AO causes the nucleus of the cells to be seen in green with organized structure nuclei. However, nonliving cells take up EtBr in the absence of membrane integrity and are seen in a range of yellow-orange-red [27]. Accordingly, cells with early apoptosis have green nuclei with condensed or fragmented chromatin and have a crescent-shaped or granular yellow-green staining. But cells with late apoptosis have orange-to-red nuclei with condensed or fragmented chromatin. Necrotic cells will show increased volume and orange-to-red organized structure nuclei [28].

**Figure 5:**
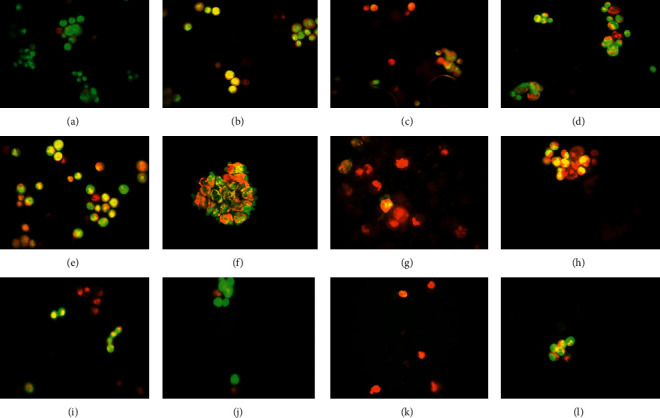
AO/EB staining assay. (a) Negative control group: the circular nucleus distributed in the center of the cell. (b) DTX treated group: nucleus showed granular appearance with yellow-green ﬂuorescence by AO staining indicating apoptosis. (c) A.m (IC50): nucleus of the cells showed orange fluorescence indicating late apoptosis. (d) A.h (IC50): the nucleus of cells showed yellow-green ﬂuorescence and located in bias. (e) A.m + DTX (IC50 + IC50) : yellow-green fluorescence was observed in the nucleus of cells. (f) A.m + DTX (IC50 + IC10) : apoptotic vesicles were observed out of the cells clearly. (g) A.m + DTX (IC25 + IC25) : the dominant red color by EB staining indicated necrosis and they were disintegrating. (h) A.m + DTX (IC10 + IC10): late-stage apoptotic cells were detected with concentrated and asymmetrically localized orange nuclear EB staining. (i) A.h + DTX (IC50 + IC50), (j) A.h + DTX (IC50 + IC10), and (l) A.h + DTX (IC10 + IC10): apoptotic cells with nucleus showed yellow-green ﬂuorescence and were located in one side of cells. (k) A.h + DTX (IC25 + IC25): the dominant red color indicated necrosis. IC values were as follows: for A.m : IC10 = 32 *µ*g/ml, IC25 = 40 *µ*g/ml, and IC50 = 57 *µ*g/ml; for A.h : IC10 = 10 *µ*g/ml, IC25 = 33 *µ*g/ml, and IC50 = 85 *µ*g/ml; and for DTX : IC10 = 0.4 *µ*M, IC25 = 3.5 *µ*M, and IC50 = 11 *µ*M.

As shown in Figure 5, the result of fluorescence microscopy of nuclear dye stained 4T1 cells showed that, in the untreated group (negative control), the nuclei were homogeneous and round, and the cells were seen in green. Green and red nuclei with condensed chromatin were observed in the groups treated with DTX, A. m, and A. h, indicating early and late apoptosis.

In combination groups, there were more apoptosis in groups *e* (IC50 A. m + IC50 DTX) and specially *f* (IC50 A. m + IC10 DTX) and apoptotic bodies were clearly evident. In group *g* (IC25 A. m + IC25 DTX), the red color of the nuclei and the increase of cell volume indicated necrosis. In group *h* (IC10 A. m + IC10 DTX), the nuclei with orange fluorescence showed late apoptosis. In the case of A. h extract + DTX combination, groups *j* (IC50 A. h + IC10 DTX) and *l* (IC10 A. h + IC10 DTX) exhibited more apoptosis than group *i* (IC50 A. h + IC50 DTX). Group *k* (IC25 A. h + IC25 DTX) showed necrosis. These data are consistent with the results of flow cytometry.

### 3.6. Effect of DTX, A.m, and A.h Extracts and Their Combinations on Wnt/*β*-Catenin Pathway Gene Expression

*β*-Catenin expression decreased in A. m, A. h, DTX, and A. h + DTX groups compared to control, but this decrease was significant in A. h + DTX (IC50 + IC25) group.

FZD7 expression in A. m + DTX (IC50 + IC25) group showed a significant decrease, but, in A. h + DTX (IC50 + IC25) group, there was a significant increase. The expression of VEGF-a did not change significantly in any of the groups.

**Figure 6:**
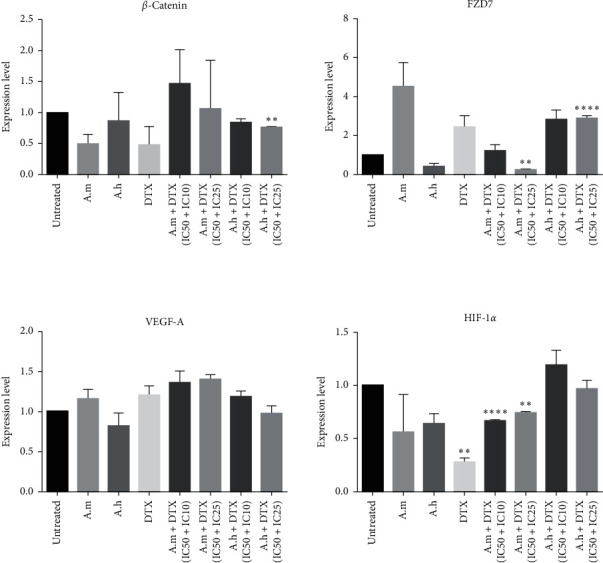
Effect of DTX, A.m, and A.h extracts and A.m or A.h + DTX combination groups on expression levels of *β*-Catenin, FZD7, VEGF-a, and HIF-1*α* genes accomplished by real-time PCR. Data are represented as the relative expression level compared to GAPDH on the basis of Pfaffl method, presented as mean ± SD. Significance was set at ^*∗*^*P* < 0.05. IC values were as follows: for A.m : IC10 = 32 *µ*g/ml, IC25 = 40 *µ*g/ml, and IC50 = 57 *µ*g/ml; for A.h : IC10 = 10 *µ*g/ml, IC25 = 33 *µ*g/ml, and IC50 = 85 *µ*g/ml; and for DTX : IC10 = 0.4 *µ*M, IC25 = 3.5 *µ*M, and IC50 = 11 *µ*M.

The expression of HIF-1*α* increased in A. h + DTX (IC50 + IC10) group, but this increase was not significant. The combination treatment in A. m + DTX (IC50 + IC10) and (IC50 + IC25) groups showed a significant downregulation in mRNA expression levels of HIF-1*α* compared to the control. Data are shown in Figure 6.

## 4. Discussion

In this *in vitro* study, we investigated the combined effect of A. m and A. h extracts with DTX-based chemotherapy on the growth suppression of 4T1 cancer cell line. The results of this study with A. m and A. h extracts showed acceptable IC50 values in inhibiting the growth of 4T1 cancer cells including 57 *µ*g/ml and 85 *µ*g/ml, respectively.

A. m as a highly valuable plant has numerous secondary metabolites and flavonoids and has shown many medicinal properties [29]. Over the past years, numerous experimental studies have demonstrated that diethyl ether and petroleum ether extracts of A. m had IC50 values of 114.7 *µ*g/ml and 105.7 *µ*g/ml against MCF7 human breast cancer cell line [16]. A. m extract showed IC50s > 100 *µ*g/ml against A549, MCF7, HepG2, HT-29, and MDBK cell lines [30].

Synergy is a process in which the combined effect of the participating elements is greater than the sum of their individual effects. Examining these interactions is important because it may have advantages for treatment, even in complex diseases such as cancer [31]; for example, ginseng increases the cytotoxicity of docetaxel, cisplatin, and doxorubicin [32].

In our study, A. m at concentrations below 100 *µ*g/ml, in combination with the DTX, showed a synergistic behavior and reduced the IC50 value of DTX with a significant and steep gradient. A. m at concentrations above 100 *µ*g/ml alone showed a higher suppression of cancer cell growth, so the CompuSyn software did not offer synergy in comparison to individual and combined modes. A. m extract also inhibited the migration of 4T1 cells both alone and in combination with DTX. Additionally, we found the increased percentages of apoptotic cells in A. m + DTX (IC50 + IC25 and IC50 + IC10) combinations compared to A. m extract alone, which were close to the percentage of apoptosis caused by DTX. In A. m + DTX (IC50 + IC10) group, apoptotic bodies were also clearly seen. It appears that when the amount of A. m extract in combination therapy is greater than DTX, the amount of apoptotic cells increases. However, A. m + DTX (IC10 + IC10) combination group showed the least necrosis, and when the amount of DTX increased, the necrosis also was increased. The A. m + DTX (IC50 + IC10 and IC50 + IC25) combination groups also significantly reduced the expression level of the HIF-1***α*** gene. It seems that IC10 of DTX in combination with IC50 of A. m was more effective than IC25 of DTX. Also, A. m + DTX (IC50 + IC25) group downregulated the expression of FZD7 gene. In other studies, Lupeol from A. m extracts reduced mRNA expression level of p53, caspase-3, and bax genes in MCF-7 and MDA-MB-231 cell lines; however, bcl-2 gene expression was decreased [22].

Docetaxel is generally accepted as the chemotherapy agent for patients with advanced breast cancer [7], but its clinical efficacy can be compromised by serious related side effects. So, controlling DTX therapy-related side effects may improve clinical outcomes in patients undergoing chemotherapy. Considering that A. m extract in combination with DTX showed synergistic behavior in concentrations below 100 *µ*g/ml, this combination may be inhibiting cancer cell growth in breast cancer and reduce the side effects of chemotherapy. According to previous reviewed information, A. m has got great scope for phytochemical and pharmacological studies to prove its therapeutic potential [19]. In combination with DTX, A. m in concentrations below 100 *µ*g/ml can be a potential complementary therapeutic option in breast cancer.

A. h fruits, oil, and even leaves in traditional medicine have been used for numerous treatments, but the efficacy of the extract in breast cancer has not been studied. A. h, though alone, had an acceptable IC50 against 4T1 cells but showed a contradictory behavior in combination therapy. In the combination of A. h with DTX, CI > 1 was obtained, indicating antagonism. Although it was able to reduce the IC50 value of DTX, it was, of course, less steep than that of A. m. A. h + DTX (IC50 + IC10 and IC10 + IC10) combination groups induced apoptosis even more than DTX alone, and A. h + DTX (IC50 + IC10) group presented 63.3% apoptosis. The gene expression analysis showed that A. h + DTX (IC50 + IC25) significantly downregulated *β*-Catenin. However, A. h + DTX (IC50 + IC25) increased expression of FZD gene; this group showed the least apoptosis compared to other groups. The good potential of A. h in inhibiting the migration of 4T1 cells and inducing apoptosis was quantitatively and qualitatively evident. Since A. h in combination mode slowly reduced the IC50 value of DTX, the synergistic behavior could be observed probably by increasing the treatment duration or changing the concentrations. In fact, the concentrations or treatment time we used for this study may not have been adequate or sufficient to create a synergistic state for A. h.

## 5. Conclusion

Briefly, we investigated the combination effect of A. m and A. h extracts with DTX for the first time. A. m and A. h extracts inhibited the growth of 4T1 cancer cells. Having definitive synergistic effect in concentrations below 100 *µ*g/ml, A. m extract can be a good alternative in combination with DTX. Natural products are a huge source of diverse elements in drug research. These types of studies help to identify susceptible plants in this context and develop these research studies. A. m and A. h extracts may have a good potential for becoming drugs given their acceptable IC50s in inhibiting the growth of 4T1 cancer cells. Nevertheless, it is necessary to examine the effects of these medicinal plants individually and in combination with DTX in animal model and clinical trial.

## Figures and Tables

**Table 1:** Name of genes, accession numbers, and nucleotide sequences of the primers used for real-time PCR.

Gene	Accession no.	Forward primer (5′-3′)	Reverse primer (3′-5′)	Size of product
HIf-1*α*	XM_017314961.1	CTGGATGCCGGTGGTCTA	ACCCCATGTATTTGTTCACGTT	148
FZD7	NM_008057.3	ATATCGCCTACAACCAGACCAT	AGGAACGGCACGGAGGAA	192
VEGFa	NM_001025257.3	GAGCAGAAGTCCCATGAAGTGA	CACAGGACGGCTTGAAGATGT	133
*β*-Catenin	NM_007614.3	ATGCGTTCTCCTCAGATGGTGTC	CAGAATCCACTGGTGAACCAAGC	187
GAPDH	NM_008084.3	CAGCCTCGTCCCGTAGACAA	GCCGTGAGTGGAGTCATACTG	177

**Table 2:** The doses for extracts (A.m and A.h) and DTX, as well as the values of CI obtained from the CompuSyn software analysis.

Combination of A.m + DTX				Combination of A.h + DTX			
Dose of A.m (*µ*g/ml)	Dose of DTX (*µ*M)	Effect	CI	Dose of A.h (*µ*g/ml)	Dose of DTX (*µ*M)	Effect	CI
15	0.8	0.49	0.32	31	1	0.1	>100
15	20	0.57	0.78	31	10	0.27	66.84
15	100	0.98	0.03	31	100	0.93	0.19
31	4	0.52	0.73	62	0.1	0.015	>100
31	20	0.6	0.73	62	1	0.11	>100
31	100	0.98	0.08	62	10	0.32	24.74
57	0.8	0.63	0.69	62	100	0.9	0.43
57	4	0.59	0.82	84	10	0.25	>100
57	100	0.98	0.15	84	100	0.91	0.56
62	0.8	0.66	0.71	125	10	0.057	>100
62	4	0.63	0.79	125	100	0.95	0.70
62	100	0.98	0.16	250	0.1	0.82	2.07
125	0.8	0.72	1.26	250	1	0.77	2.26
125	4	0.65	1.48	250	10	0.39	10.00
125	20	0.69	1.40	250	100	0.95	1.40

## Data Availability

The data supporting the findings of this study are available within the article.

## Supplementary Materials

Supplementary Materials Table 1: scientific names of selected plants for screening in this study, herbarium code, part of the plant used in the study, and *in vitro* cytotoxicity of their hydroalcoholic extracts.
