# Supplementary material for: A Comparative Study on Anticancer Effects of the Alhagi maurorum and Amygdalus haussknechtii Extracts Alone and in Combination with Docetaxel on 4T1 Breast Cancer Cells
Source: Evid Based Complement Alternat Med. 2021 Jun 14;2021:5517944. doi: 10.1155/2021/5517944 (PMC8219415; doi:10.1155/2021/5517944)
Supplement: Supplementary Materials — Table 1: scientific names of selected plants for screening in this study, herbarium code, part of the plant used in the study, and in vitro cytotoxicity of their hydroalcoholic extracts. [file 5517944.f1.docx]

Table 1. Scientific names of selected plants for screening in this study, herbarium code, part of the plant used in the study, and *in-vitro* cytotoxicity of their hydro-alcoholic extracts.

| No. | Family | Plant species | Herbarium code | Part of the plant used in this study | Cytotoxicity IC50 (µg ml^-1^) on 4T1 cell line |
| --- | --- | --- | --- | --- | --- |
| 1 | Apiaceae (Umbelliferae) | Echinophora Cinerea (Boiss.) | 217 | Arial parts | 200 |
| 2 | Apiaceae (Umbelliferae) | Echiniphora platyloba Dc. | 249 | Arial parts | 477 |
| 3 | [Solanaceae](https://en.wikipedia.org/wiki/Solanaceae) | Physalis alkekengi | 153 | Fruit | 231 |
| 4 | Lamiaceae | Ziziphora Clinopodioides Lam | 253 | Leaves | 220 |
| 5 | Brassicaceae | Cardaria draba (L.) Desv. | 257 | Arial parts | >500 |
| 6 | Fabaceae | **Alhagi maurorum**  **(Alhagi Persarum Boiss& Buhse)** | 472 | Arial parts | **57** |
| 7 | Lamiaceae | Teucrium Polium L. | 522 | Arial parts | 238 |
| 8 | Apiaceae | Falcaria vulgaris Brenh. | 490 | Arial parts | 166 |
| 9 | [Malvaceae](https://www.uibk.ac.at/botany/staff/publikationen/schoenswetter_peter/58.pdf) | Alcea koelzi I.Riedl | 139 | Flowers | >500 |
| 10 | Scrophulariaceae | Verbascum songaricum Schrenk ex Fisch.& C.A.mey | 251 | Flower heads | 367 |
| 11 | Apiaceae | Eryngium billardieri Del. | 609 | Arial parts | 393 |
| 12 | Rosaceae | **Amygdalus Haussknechtii (C.K.Schneider) Bornm.** | 534 | Leaves | **85** |
| 13 | Asteraceae | Cichorium intybus | 148 | Flower heads | 141 |
| 14 | Equisetaceae | Equisetum arvense L. | 516 | Arial parts | 452 |
| 15 | Solanaceae | Hyoscyamus niger | 402 | Seeds | 227 |
| 16 | Fagaceae | Quercus infectoria | 124 | Fruits | 160 |
| 17 | [Asteraceae](https://en.wikipedia.org/wiki/Asteraceae) | Helianthus annuus | 702 | Petals | >1000 |
| 18 | Lamiaceae | Salvia officinalis | 663 | Arial parts | 204 |
| 19 | Lamiaceae | Hyssopus officinalis L. | 177 | Arial parts | 181 |
| 20 | Asteraceae | Tanacetum parthenium (L.) Schultz- Bip. | 111 | Arial parts | 189 |
